# Morphometric Evaluation of Preeclamptic Placenta Using Light Microscopic Images

**DOI:** 10.1155/2014/293690

**Published:** 2014-06-23

**Authors:** Rashmi Mukherjee

**Affiliations:** School of Medical Science and Technology, Indian Institute of Technology, Kharagpur, West Bengal 721302, India

## Abstract

Deficient trophoblast invasion and anomalies in placental development generally lead to preeclampsia (PE) but the inter-relationship between placental function and morphology in PE still remains unknown. The aim of this study was to evaluate the morphometric features of placental villi and capillaries in preeclamptic and normal placentae. The study included light microscopic images of placental tissue sections of 40 preeclamptic and 35 normotensive pregnant women. Preprocessing and segmentation of these images were performed to characterize the villi and capillaries. Fisher's linear discriminant analysis (FLDA), hierarchical cluster analysis (HCA), and principal component analysis (PCA) were applied to identify the most significant placental (morphometric) features from microscopic images. A total of 10 morphometric features were extracted, of which the villous parameters were significantly altered in PE. FLDA identified 5 highly significant morphometric features (>90% overall discrimination accuracy). Two large subclusters were clearly visible in HCA based dendrogram. PCA returned three most significant principal components cumulatively explaining 98.4% of the total variance based on these 5 significant features. Hence, quantitative microscopic evaluation revealed that placental morphometry plays an important role in characterizing PE, where the villous is the major component that is affected.

## 1. Introduction

Preeclampsia (PE), a pregnancy specific vasoconstrictive condition, is defined as the occurrence of hypertension and significant proteinuria in a previously healthy woman on or after the 20th week of gestation [1]. Major maternal complications include disseminated coagulopathy, acute renal failure, liver impairment, pulmonary oedema, and seizures (eclampsia). PE increases maternal long-term health risks of later hypertension, stroke, and ischemic heart disease [2, 3]. There is growing evidence that women with a previous history of PE tend to develop metabolic syndromes several years later, including insulin resistance, dyslipidemia, higher blood pressure, body mass index (BMI), and leptin levels [4, 5]. Babies born to preeclamptic mothers are also affected; a third are born preterm, 20% are growth restricted, and evidence indicates that there may be an increase of three- to ten-fold in perinatal deaths [6, 7]. PE remains a considerable public health threat in both developed and developing countries, leading to maternal and perinatal morbidity and mortality worldwide. World Health Organization (WHO) estimates that over 100,000 deaths occur annually due to PE worldwide affecting nearly 2–8% of pregnancies in the developed countries [8]. However, the impact of the disease is felt more severely in developing countries where its prevalence rate reaches nearly 16.7%.

Despite decades of intense research, PE remains a significant challenge to medical science due to the continuing mystery of its etiology and unpredictable nature [9]. Although the exact cause of PE remains unknown, it is generally accepted that deficient trophoblast invasion, generalized endothelial dysfunction, and anomalies in placental development lead to PE [10]. In PE, inadequate extravillous trophoblast invasion into the placental bed during early placental development results in failure of the physiological conversion of the spiral arteries that is characteristic of normal pregnancy [11]. Spiral arteries remain narrow and blood supply to the fetus is restricted, leading to deficient uteroplacental circulation and a poorly perfused, ischemic, and hypoxic placenta [12, 13]. The poorly perfused and hypoxic placenta releases increased amounts of vasoactive factors, disrupts syncytial architecture, and contributes to endothelial cell dysfunction. As such, in this ischaemic environment, structural compensations of the villi such as increased surface area or decreased diffusion distance may occur. Alternatively, there may be increased damage such as fibrotic deposition or necrotic areas.

Few attempts have been made for comparative studies of preeclamptic versus normotensive placental morphology. Mayhew reported that villous surface area alters disproportionately to volume in preeclamptic pregnancies [14]. Resta et al. observed significant hyperramification of the capillary loops in terminal villi of preeclamptic placentae along with abnormal vessel profiles and narrow vessel lumina [15]. Increased prevalence of inflammation, infarction, ischaemia, haemorrhage, and syncytial knots in preeclamptic placentae is also reported [16]. However, contradictory reports exist which do not associate PE with any main or interaction effects on placental morphometry [17, 18].

Interrelationship between placental function and morphology with that of PE pathogenesis is yet to be explored. The present study investigates the placental villous and capillary parameters in preeclamptic and normotensive pregnancies which could affect the haemodynamics of maternofetal exchange. Quantitative characterization based on morphometric analysis of histological placental images has been performed to identify significant pathological signatures of preeclamptic placentae.

## 2. Materials and Methods

**Figure 1:**
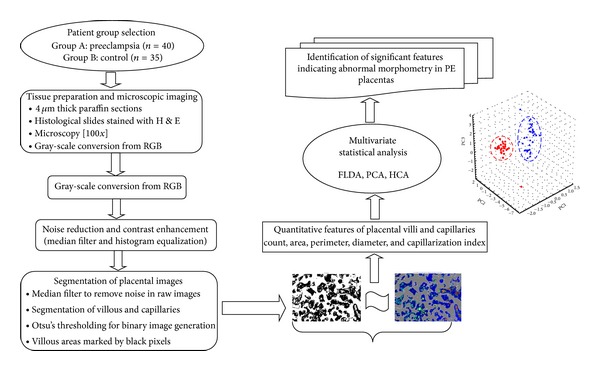
Overview of the study design.

The schematic overview of the present study is given in Figure 1.

### 2.1. Subject Selection

The study was conducted at Medipath Clinic, Paschim Midnapur, India. Following informed consent, placental tissues were taken from 45 preeclamptic cases (Group *A*) and 35 normotensive pregnant women after delivery (Group *B*). Diagnosis of PE was made according to the following criteria:* de novo* appearance of hypertension (SBP ≥140 mmHg or DBP ≥90 mmHg) and proteinuria (≥0.3 g/24 h of urinary protein or ≥2 + reading on a dipstick) after the 20th week of gestation in normotensive women [19].

Groups were matched for maternal age and parity. It was ensured that all women had normal liver function tests and were euthyroid with BMI <25 and with no evidence of any other endocrinopathy. Woman suffering from any other preexisting medical conditions like diabetes mellitus, ischemic heart disease, stroke, peripheral vascular disease, cardiac, renal, hepatic dysfunction, chronic hypertension, preexisting seizure disorder, eclampsia, pregestational diabetes, placental abruption, gestational diabetes, thyroid disease, and dyslipidaemia were excluded.

### 2.2. Sample Preparation

Placental tissue samples were collected after termination of pregnancy and washed in phosphate buffered saline (PBS) to remove blood contamination. After fixing in 10% neutral buffered formalin (NBF) overnight, placental tissues were processed to obtain 4 *μ*m thick paraffin sections on poly-L-lysine (Cat. number P8920 Sigma-Aldrich, St. Louis, MO, USA) coated glass slides for histological studies. Sections were deparaffinized, rehydrated in a graded series of ethanols, and stained with hematoxylin and eosin (H&E). Stained sections were dehydrated in a series of ethanols and mounted using DPX (product code: 042848 Sisco Research Laboratories-SRL, Pvt. Ltd., Mumbai, India).

### 2.3. Microscopic Imaging

Numerous photomicrographs were optically grabbed by Zeiss Observer.Z1 Microscope (Carl Zeiss, Germany) under 10x objective (NA 0.25) in RGB (red, green, and blue) colour space. Each pixel size of the grabbed digital images was 0.63 *μ*m. each image size was taken as 1388 × 1040 using the software Axiovision (version 4.7.2, Carl Zeiss) image analysis package.

### 2.4. Image Preprocessing and Segmentation of Villous and Capillaries

**Figure 2:**
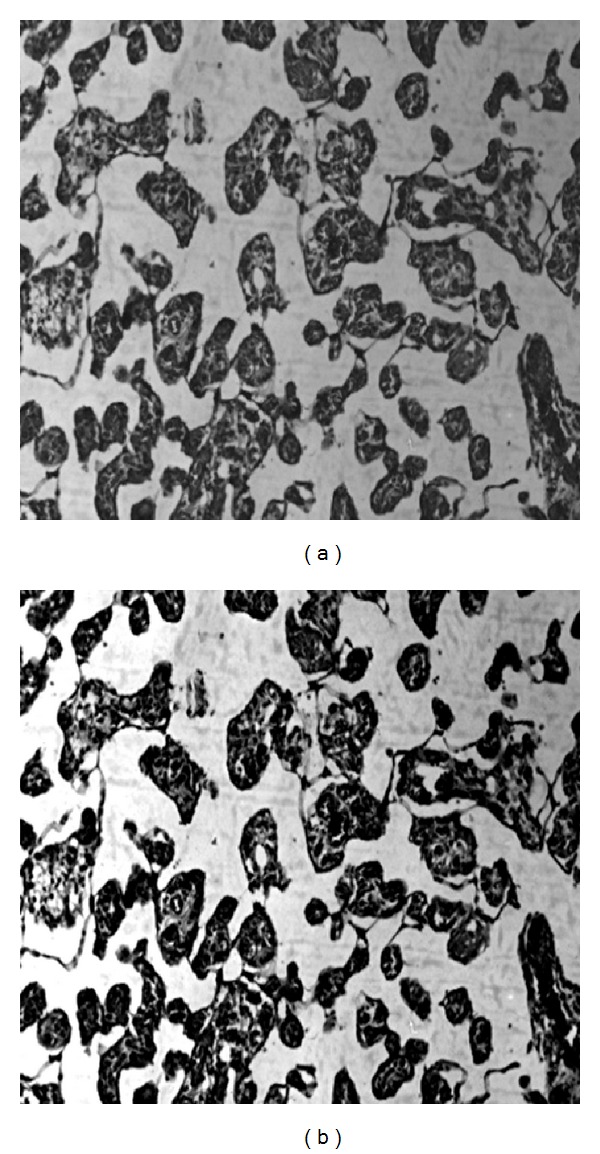
Microscopic image of placenta (a) original intensity image; and (b) contrast enhanced image.

The RGB images of placental tissue sections were converted into gray scale based on a weighted sum of R, G, and B components [0.2989 ∗ R + 0.5870 ∗ G + 0.1140 ∗ B] using “rgb2gray” command of MATLAB software. In order to reduce the noise level due to staining variation, median filter was used and, simultaneously, the image contrast was enhanced using histogram equalization technique (Figures 2(a) and 2(b)).

**Figure 3:**
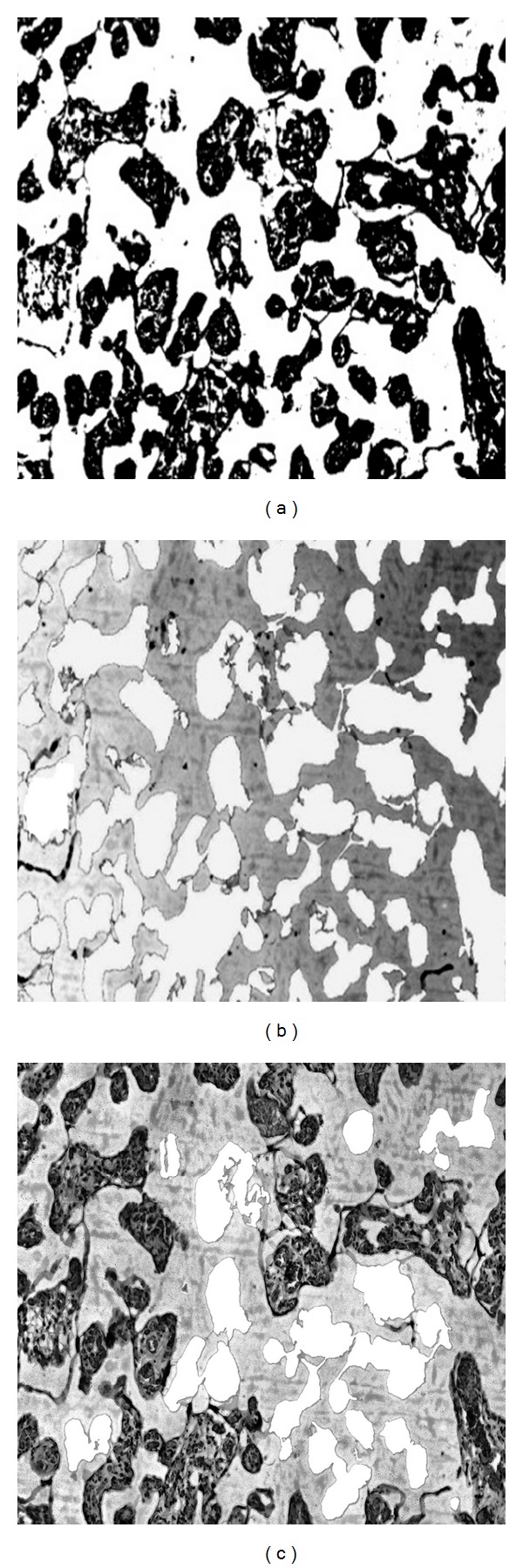
(a) Binary image obtained by Otsu's thresholding; (b) superimposing the binary image for the demarcating villous region; (c) superimposing the binary image for selection of complete villi.

*Villous Area Segmentation.* The regions of clinical importance to be segmented were villous and its blood capillaries area. Here, Otsu's thresholding using “graythresh” command of MATLAB software was performed for converting gray scale image into its binary one [20]. This method yielded a bimodal histogram (background and villous regions) where threshold was selected in its valley. Hence, the villous areas were extracted by considering the pixel intensities higher than the threshold. Here villous pixels were represented by “1”s (Figures 3(a)–3(c)). Here we observed that the villous regions contained unwanted islands inside them, which in some cases were due to the capillaries present or other structures in the tissue. So, these unwanted islands were removed by mask-based filtering and then morphological processing. 

**Figure 4:**
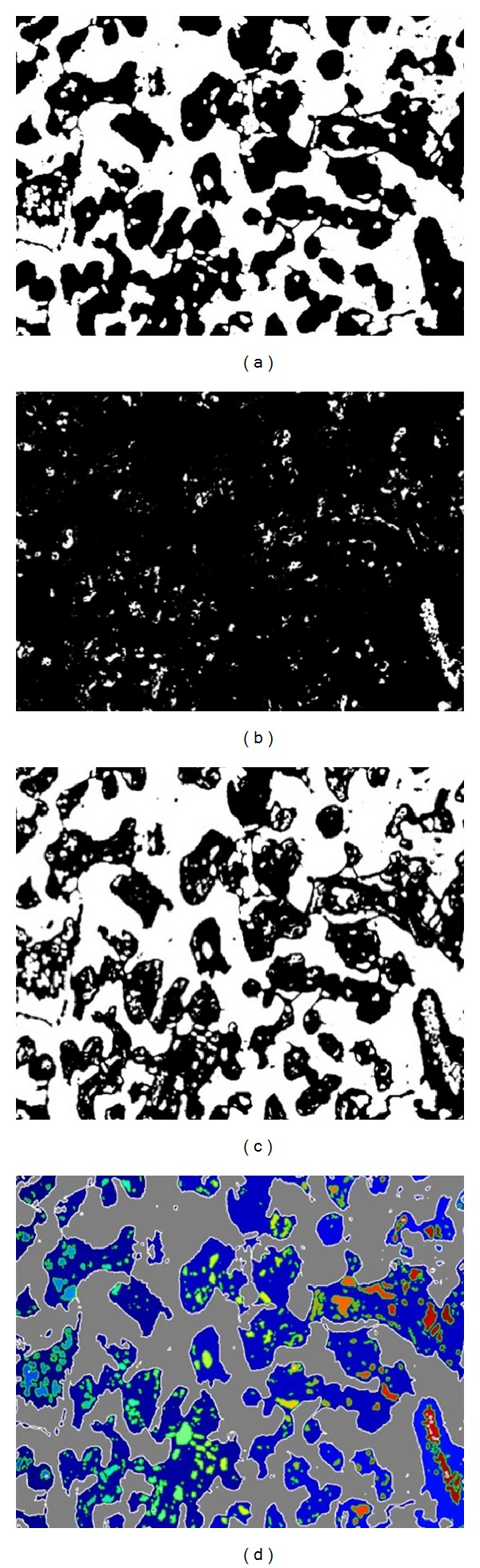
Segmentation (a) capillary extraction, which uses morphological operations to extract the clearly visible capillaries; (b) result for capillary extraction showing the 3rd channel of the Lab colour space; (c) final result of blood capillary segmentation by combining (a) and (b) where white regions inside the black villi are the capillaries; and (d) final result with marked regions. Regions with white boundaries indicate villi and the ones with green boundaries indicate blood capillaries.

*Blood Capillary Segmentation.* Morphological operations like dilation, erosion, opening, and a structuring element were used to segment the visible capillaries. A structuring element was here used to obtain shape/spatial orientation of capillaries inside the segmented villous regions where its kernel size was 3 × 3. Segmented capillaries filled with blood were extracted using color deconvolution and colour space transformation. Color deconvolution is based on the separation of different stains for that tissue which produces different colours for different regions (Figures 4(a)–4(d)).

### 2.5. Quantitative Features of Placental Villi and Capillaries

Quantitative characterization of placental villi and capillaries is vital for a better understanding of its morphology and function in physiological and pathological condition. Here the geometric features, namely, count, area, perimeter, and diameter, were extracted from the segmented villi and capillaries of the placental images as follows.

(i) *Villous count:* this corresponds to total number of villous regions in all grabbed images.
(ii) *Villous area:* area is the total number of pixels present in the segmented villous regions; that is,
  (1) $$\begin{array}{c} \text{Area}=\underset{x}{\sum }\underset{y}{\sum }\;f\left(x,y\right), \end{array}$$
  where *f*(*x*, *y*) represents the pixel value corresponding to the (*x*, *y*) position of binary villous image *f*(*x*, *y*) = 1; if *f*(*x*, *y*)∈ object else *f*(*x*, *y*) = 0.
(iii) *Villous perimeter:* it can be defined as the number of boundary pixels present in the villous region; that is, Perimeter = ∑_*x*_∑_*y*_ *f*(*x*, *y*), where *f*(*x*, *y*) = 1; if *f*(*x*, *y*)∈ villous boundary only else *f*(*x*, *y*) = 0.
(iv) *Villous diameter:* villous diameter was calculated using the formula:
  (2) $$\begin{array}{c} \sqrt{4*\frac{\text{Area}}{\pi }}. \end{array}$$
(v) *Capillary count:* it was computed by total number of capillaries in one image.
(vi) *Capillary area:* area of each individual capillary was obtained by considering total number of pixels present in the capillary region.
(vii) *Capillary perimeter:* perimeter of each individual capillary was computed by estimating total number of boundary pixels surrounding the capillary region.
(viii) *Capillary diameter:* capillary diameter was calculated using
  (3) $$\begin{array}{c} \text{Capillary}\;\text{diameter}=\sqrt{4*\frac{\text{Area}}{\pi }}. \end{array}$$
(ix) *Capillarization index (%):*
  (4) $$\begin{array}{c} \text{CI}=\frac{\text{Total}\;\;\text{area}\;\;\text{of}\;\;\text{capillaries}}{\text{Total}\;\;\text{area}\;\;\text{of}\;\;\text{villi}\;\;\text{in}\;\;\text{image}}*100. \end{array}$$
(x) *Capillarization index per villous (%):*
  (5) $$\begin{array}{c} \text{CI}\;\left(\text{villous}\right)=\frac{\text{The}\;\;\text{total}\;\;\text{area}\;\;\text{of}\;\;\text{capillaries}}{\text{Area}\;\;\text{of}\;\;\text{parent}\;\;\text{villous}}*100. \end{array}$$

### 2.6. Statistical Analysis

Data are presented as mean ± SD. Data were compared using Student's *t*-test wherever appropriate. *P* ≤ 0.05 was considered to be significant. Results were statistically analyzed using KyPlot version 2.0 beta 13 software (software developed by Koichi Yoshioka), SPSS software (version 11; SPSS, Inc., Chicago, IL), and MATLAB 7.1.0 (service pack 3, Mathworks Inc., USA).

#### 2.6.1. Discriminant Analysis

Fisher's linear discriminant analysis (FLDA) was used to identify the most useful set of morphological features that segregate the two groups [21]. It is one of the important multivariate techniques commonly used to discriminate study groups by projecting high-dimensional data onto a linear function. The weights of this function are obtained by maximizing the ratio of between groups variability to within groups variability, which is often called Fisher's criterion. Let us consider the *d*-dimensional features for discriminating two groups *A* and *B* on the basis of the collected samples. In doing this, Fisher's discriminant function is defined as the linear function *w* ^*T*^ *x* that maximizes the criterion

(6) $$\begin{array}{c} J\left(w\right)=\frac{w^{T}S_{B}w}{w^{T}S_{W}w}, \end{array}$$

where *w*, *S*_*B*_, and *S*_*W*_ denote weights, between and within variances of two groups, respectively. By maximizing  *J*(*w*), the optimal weights are found as

(7) $$\begin{array}{c} w=S_{\text{pooled}}^{-1}\left(\overset{-}{x_{1}}-\overset{-}{x_{2}}\right), \end{array}$$

where $\overset{-}{x_{1}}$ and $\overset{-}{x_{2}}$ indicate the samples means of groups *A* and *B* under consideration. Hence, Fisher's linear discriminant function is given by

(8) $$\begin{array}{c} y={\left(\overset{-}{x_{1}}-\overset{-}{x_{2}}\right)}^{T}S_{\text{pooled}}^{-1}x. \end{array}$$

FLDA assisted in evaluating the morphometric features based on overall classification accuracy.

#### 2.6.2. Multivariate Analysis

The overall grouping structure based on morphometric features was visualized using the hierarchical cluster analysis (HCA). HCA is a simple and systematic clustering technique for hierarchically grouping the objects that are “close” to one another. The “closeness” criterion between any two objects is computed based on the Euclidean distance measure repeatedly. As such, similar objects are grouped together when the distance is the least. The output of such iterative process is graphically represented by dendrogram. The number of clusters depends on the threshold selected by the decision maker.

Subsequently, principal component analysis (PCA) was performed to provide further information on how these morphometric features cause such groupings in HCA based on FLDA scores. PCA, a well-known multivariate data reduction technique, projects the correlated higher dimensional data space into uncorrelated lower dimensional components using an orthogonal transformation. The principal components are chosen according to their significance with respect to explaining maximum variance [21].

## 3. Results and Discussion

The clinical characteristics of the preeclamptic and normotensive pregnant women are shown in Table 1. Blood pressure parameters, namely, systolic (*P* < 0.001) and diastolic (*P* < 0.001), were significantly different between the two groups. The gestational age at sampling (*P* < 0.001) was significantly lower in the PE group as compared to normotensive cases.

Morphometric features of the placental images are summarized in Table 2. It can be observed that the villous parameters (count, area, perimeter, and diameter) are significantly increased in PE as compared to controls (*P* < 0.001). Capillary areas as well as its index are significantly increased in the preeclamptic group. These findings are in good agreement with a similar study of Ducray et al. which reported an increased density of the villous arrangement in the preeclamptic group [11]. This may be attributed to the increased growth or hyperramification of the placental villi. As such, increased density of the villous arrangement can reduce the intervillous space, thus impacting blood circulation in this area. Even though the villous surface area may be increased, however, blood flow can decrease, thereby, compromising maternofetal exchange. Figures 7(a) and 7(b) show H&E photomicrographs from a typical preeclamptic placenta illustrating the morphological features which were found to be significantly different by morphometry, compared to a normal placenta of identical gestational age.

FLDA was then used to identify the most useful features using classification accuracy based ranking strategy that segregate the groups with respect to all the significant morphometric features. This approach permitted filtering out of significant features which were actually differentiating the placental morphology amongst the two groups (Table 3). The classification accuracy allowed identification of five highly significant morphometric features (viz., villous count, villous area, villous perimeter, villous diameter, and capillarization index) leading to more than 90% overall accuracy that were prominent between the groups.

### 3.1. Multivariate Analysis

**Figure 5:**
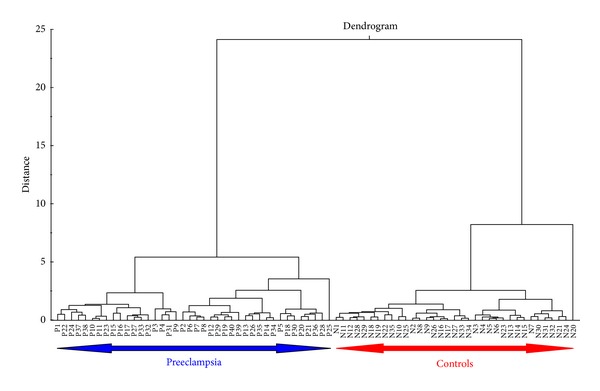
HCA of prominent significant placental morphometric features.

**Figure 6:**
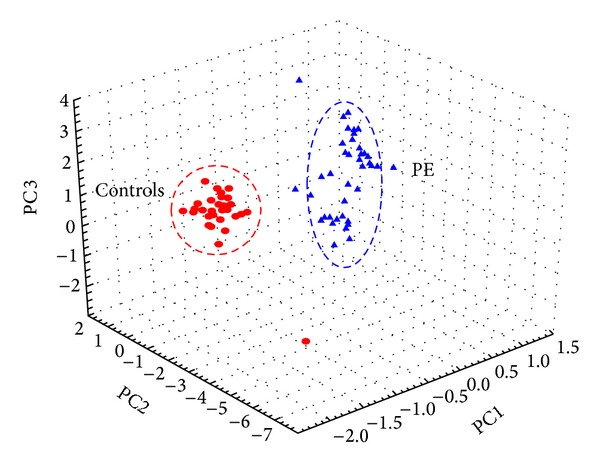
PCA of prominent significant placental morphometric features.

**Figure 7:**
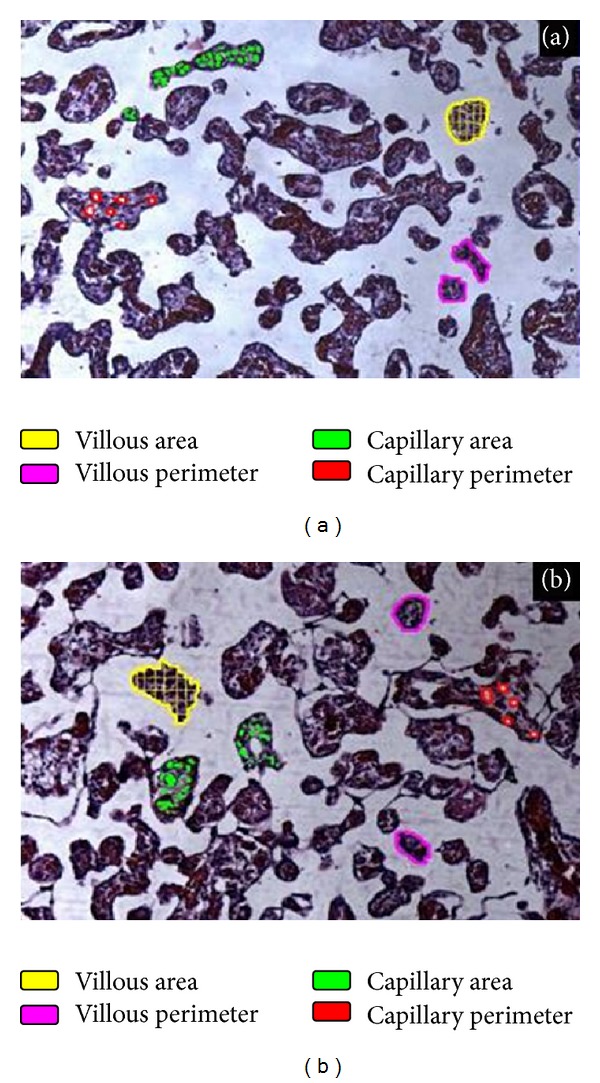
H&E photomicrograph from (a) a typical normal placenta illustrating the morphological features which were found to be significantly different by morphometry, compared to (b) a preeclamptic placenta of identical gestational age.

Multivariate techniques were used to understand how PE and control placental samples were grouped according to their significant morphometric features. The HCA based dendrogram for all 75 placental samples is shown in Figure 5. Two large subclusters are clearly visible in the dendrogram suggesting that these features segregated PE and control groups efficiently. Working from left to right, the first major subcluster contains all the PE cases followed by the control cases. In order to avoid multicollinearity issue, PCA was used here to compress five significant morphometric features. It returned three most significant principal components (PCs) to cumulatively explain 98.4% of the total variability present in the data; where PC1, PC2, and PC3 explained 83.5%, 10.8%, and 4.1% of the same, respectively. These three PCs lead to a better discrimination of PE and control groups as shown in 3D scatter plot (see Figure 6).

Studies involving placental villous morphometry have focused on the peripheral (intermediate and terminal) villi, as these villi form the surface for actual maternofetal exchange. Reductions in placental villous and vascular morphology in pregnancies complicated with PE have also been previously reported [22]. Significant hyperramification together with irregular profiles and narrow lumina was observed in the capillary loops of terminal villi of preeclamptic placentae [15]. In order to know whether the observed alterations in placental morphometry of PE cases were due to changes of gestational age rather than the diseased condition itself, a subgroup analysis was further performed by comparing only those PE cases with controls possessing the same weeks of gestation. The results are summarized in Table 4. It can be seen that all the earlier identified parameters (Table 3) are significantly altered in PE as compared to normotensive pregnant women. However, it is to be stated here that the sample size for the gestational matched cases was still relatively small, which limited the statistical power to achieve a definitive conclusion. Future studies should be performed using large sample numbers to establish a more definitive conclusion. There were many preterm births for PE cases in the present study as compared to controls. Another limitation of the study was that the control group mainly consisted of women delivering at term. Comparing the placental morphometry in PE patients delivering at term versus those PE patients delivering at preterm and also with normotensive women who delivered prematurely may help to elucidate the underlying pathologic process.

It is known that in preeclamptic patients delivering at term, placental histomorphometry results are comparable to those of normotensive control subjects [23]. It is attributed to the fact that, in these cases, a compensatory mechanism may exist which protects uteroplacental blood flow. On the other hand, this protective mechanism is lacking in PE cases with preterm delivery [24]. Moreover, early- and late-onset PE is now being considered as two different pathophysiological disorders, with overlapping clinical signs. Recently, it has been shown that early-onset PE has a larger impact on the fetus, neonate, and unfavorable long-term maternal consequences than late-onset cases [21]. Further studies looking into the placental morphometry of these two subsets of PE can provide a better insight into the role of placental pathology for the diseased condition.

## 4. Conclusions

Summarizing evaluations of the morphometrical features showed significant difference in the number and structure of the villous region in PE. These results suggest a reduced villous carrying capacity, thereby affecting placental transport and haemodynamics, compounding the ischaemia resulting in reduced maternal spiral artery flow. Further studies are required to examine whether these features observed are associated with the time of onset of gestational hypertension.

## Figures and Tables

**Table 1:** Clinical characteristics of women with PE and controls.

Parameters	PE (*n* = 40)	Controls (*n* = 35)	*P* value
Age (years)	28.4 ± 4.7	27.5 ± 4.2	0.3876 NS
SBP (mm/Hg)	152 ± 4.9	116 ± 8.2	0.00009 *S
DBP (mm/Hg)	92 ± 6.7	74 ± 4.3	0.00003 *S
Gravidity	1.89 ± 0.59	1.79 ± 0.74	0.5174 NS
BMI (kg/m^2^)	24.1 ± 3.7	23.9 ± 4.2	0.8271 NS
Weeks of gestation (wks)	34.6 ± 2.5	37.9 ± 2.2	0.0002 *S

Results are expressed as Mean ± SD; SBP: systolic blood pressure; DBP: diastolic blood pressure; BMI: body mass index; *P value ≤ 0.001; S: significant; NS: not significant.

**Table 2:** Morphometrical features of women with PE and controls.

Features	PE (*n* = 40)	Controls (*n* = 35)	*P* value
Villous count	188.5 ± 16.9	128.8 ± 9.2	0.00003 *S
Villous area	4018.7 ± 25.7	3028 ± 34.7	0.00002 *S
Villous perimeter	267.5 ± 12.1	187.1 ± 7.9	0.00012 *S
Villous diameter	127.625 ± 7.5	107.6 ± 3.1	0.00008 *S
Capillary count	665.8 ± 28.1	509.2 ± 16.6	0.00015 *S
Capillary area	155.8 ± 13.6	132.6 ± 15.1	0.00016 *S
Capillary perimeter	34.9 ± 3.7	35.1 ± 3.8	0.8183 NS
Capillary diameter	16.8 ± 1.9	16.2 ± 1.7	0.1563 NS
Capillarization index (%)	15.7 ± 0.8	13.1 ± 2.1	0.00019 *S
Capillarization index/villous (%)	2.91 ± 0.14	2.69 ± 0.07	0.00018 *S

Results are expressed as Mean ± SD; *P value ≤ 0.001; S: significant; NS: not significant.

**Table 3:** Classification results of 75 patients in PE and controls using FLDA and their ranking according to classification accuracy.

Morphometric features	Overall accuracy (%)	Rank
Villous count	98.7	1
Villous area	96.5	2
Villous diameter	96.0	3
Villous perimeter	92.4	4
Capillarization index (%)	92.3	5
Capillarization index/villous (%)	78.8	6
Capillary area	70.0	7
Capillary count	56.2	8

**Table 4:** Comparative study of morphometrical features in gestational age matched [35-36 weeks] cases.

Features	PE (*n* = 10)	Controls (*n* = 8)	*P* value
Villous count	190.2 ± 12.3	150.6 ± 14.5	0.0001 *S
Villous area	4025.4 ± 21.3	3050.8 ± 22.9	0.0001 *S
Villous perimeter	268.4 ± 10.9	185.3 ± 6.7	0.0001 *S
Villous diameter	129.78 ± 8.5	105.9 ± 4.1	0.0001 *S
Capillary count	670.8 ± 22.5	513.6 ± 13.4	0.0001 *S
Capillary area	162.3 ± 11.3	134.2 ± 9.2	0.0001 *S
Capillary perimeter	34.9 ± 1.8	35.3 ± 2.7	0.7114 NS
Capillary diameter	16.7 ± 1.4	16.5 ± 1.1	0.1372 NS
Capillarization index (%)	16.4 ± 0.3	11.9 ± 1.8	0.0001 *S
Capillarization index/villous (%)	2.94 ± 0.08	2.65 ± 0.03	0.0001 *S

Results are expressed as Mean ± SD; *P value ≤ 0.001; S: significant; NS: not significant.
